# A Systematic Review and Meta-Analysis Comparing Burn Healing Outcomes Between Silver Sulfadiazine and Aloe vera

**DOI:** 10.7759/cureus.30815

**Published:** 2022-10-28

**Authors:** Nicole J Levin, Young Erben, Yupeng Li, Tara J Brigham, Alison J Bruce

**Affiliations:** 1 Medicine, Florida Atlantic University Charles E. Schmidt College of Medicine, Boca Raton, USA; 2 Vascular and Endovascular Surgery, Mayo Clinic, Jacksonville, USA; 3 Political Science and Economics, Rowan University, Glassboro, USA; 4 Mayo Clinic Libraries, Mayo Clinic, Jacksonville, USA; 5 Dermatology, Mayo Clinic, Jacksonville, USA

**Keywords:** dermatology, plastic surgery, silver sulfadiazine, aloe vera, wound healing, burns

## Abstract

Burn wounds remain a prevalent problem in the developed and developing world. A multitude of remedies has been tested. This study evaluated the healing time of second- and third-degree burn wounds between silver sulfadiazine (SSD) and *Aloe vera* (AV). In July 2020, a systematic review of MEDLINE (Ovid) and PubMed (National Library of Medicine) was performed to identify studies that reported healing of second- and third-degree burns using either SSD or AV. Articles meeting the inclusion criteria were screened and carefully analyzed. Our goal was to report the healing time for these burns using SSD and AV. A total of eight studies published between 1988 and 2018 reporting healing of second- and third-degree burns using SSD and AV were included in this review. Six were cohort studies and two were randomized controlled trials. The studies included both animal and human subjects. The meta-analysis demonstrated that the mean time to wound healing favored AV (RR: -1.34, 95% CI: -1.8 to 0.9, p < 0.001). It would seem that time to healing benefitted those burns in which AV was utilized. In conclusion, increased consideration and emphasis should be placed on using AV to aid the healing of second- and third-degree burns.

## Introduction and background

Burn injuries pose a great burden worldwide and account for approximately 180,000 deaths per year [1]. Moreover, non-fatal burn injuries are a major cause of morbidity [1]. The goal of burn treatment is to prevent infection because infection delays wound healing [2]. The ideal topical burn treatment should reduce the wound’s bacterial load without hindering the wound-healing process. Therefore, silver sulfadiazine (SSD), a well-known bactericidal agent, is currently the gold standard in topical burn treatment and is often utilized for the treatment of second- and third-degree burns [3]. Its efficacy in wound care is based solely on its antibacterial properties. However, microbial resistance to antibacterial compounds like SSD is becoming an increasing concern [2-4]. Other undesirable side effects from SSD use have also been reported, including delayed wound healing [5-9], argyria, leucopenia, hepatic and renal toxicity, and allergic contact dermatitis [10,11].

Another well-known and widely used compound for the treatment of burns is *Aloe vera* (AV). AV belongs to the *Liliaceae* family and is indigenous to the Arabian Peninsula [12]. The gel within this medicinal plant has been utilized for centuries in many different countries for a variety of skin conditions. AV has been successfully used to prevent skin ulcers and treat burn wounds, postoperative wounds, and chronic wounds, such as pressure ulcers. AV has also been reported to heal first- and second-degree burns [13]. AV’s anti-inflammatory and anti-bacterial features contribute to its potent wound-healing properties.

AV contains 75 potentially active compounds that are responsible for its unique anti-microbial, anti-inflammatory, and growth-factor-like effects [14]. Some key constituents include mannose-6-phosphate, salicylic acid, lignin, and saponin. Mannose-6-phosphate facilitates collagen synthesis and enhances the rate of wound contraction and macrophage function [15]. Wound treatment with Aloe also increases the expression of β1-, α6-, and β4-integrin and E-cadherin in human primary epidermal keratinocytes, contributing to cell migration and wound healing [16]. Salicylic acid is responsible for some of Aloe’s anti-inflammatory effects. However, salicylic acid alone does not possess the same properties in burn wound healing [17]. AV is effective in inhibiting inflammation through the inhibition of proinflammatory cytokines (interleukin (IL)-6 and IL-8), the reduction of leukocyte adhesion, an increase of IL-10 levels, and the reduction of tumor necrosis factor-alpha (TNF-α) levels [12]. Furthermore, AV contains magnesium lactate, which can help reduce histamine synthesis, resulting in a decrease in skin itching and irritation [18]. Lignin enhances penetration of other components within Aloe and saponin confers antiseptic properties. Other antiseptic agents contained in Aloe include lupeol, urea nitrogen, cinnamic acid, phenols, and sulfur. The main component responsible for Aloe’s wound healing effects is its glycoprotein fraction and it also inhibits thromboxanes, which further improves healing [19,20]. It also increases collagen type III, which in turn accelerates wound contraction and the strength of the resulting scar tissue is increased [14].

Existing literature demonstrates that AV gel seems to be efficacious in the treatment of second-degree burns with no or less toxicity than SSD. Moreover, AV is a less expensive and more accessible option than SSD, making it a more cost-effective and convenient treatment option. To the best of our knowledge, this is the first systematic review and meta-analysis comparing wound healing outcomes between topical SSD and AV gel for the treatment of second- and third-degree burns.

## Review

Methods

Study Selection

Initial studies were identified on July 6, 2020, by a medical librarian in the PubMed and Ovid MEDLINE (1946 to present) database. There were no limits to the publication date or study design, but an English-language filter was used. The search strategy was created using a combination of keywords and standardized index terms (Table 1). Search terms included database-specific controlled vocabulary and additional free-text terms/keywords such as burns, thermal injury, Aloe, and silver sulfadiazine.

**Table 1:** Combinations of words and phrases in the literature search method

Final search MEDLINE
1. Exp burns/or (burn or burns or burned or scald*).ti,ab. or thermal injur*.ti,ab.
2. Aloe*.mp.
3. ("sulfadiazine" or " sulfanil amidopyrimidine" or " sulfanilamidopyrimidine" or "adiazine" or "aldiazine" or "coco-diazine" or "cocodiazine" or "codiazine" or "cremodiazine" or "debenal" or "di azo mil" or "diastrep" or "diazine" or "eskadiazine" or "eustral" or "keladiazine" or "liquadiazine" or "microsulfon" or "pirimal" or "pyrimal" or "sodium sulfadiazine" or "sterazine" or "sulfacombin" or "sulfadiazin" or "sulfadiazine sodium" or "sulfadiazine sulfur" or "sulfapyrimidine" or "sulfazine" or "sulphadiazine" or "sulfadiazine silver" or "aldo-silvederma" or "brandiazin" or "burnazin" or "dermazin" or "flamazin" or "flamazine" or "flammazin" or "flammazine" or "flint ssd" or "geben" or "silbecor" or "sildaflo" or "silvadene" or "silvadyn" or "silvederma" or "silver sulfadiazinate" or "silver sulfadiazine" or "silverdiazina" or "silverol" or "silvirin" or "sofargen" or "ssd af" or "ssd ointment" or "sterizol" or "sulfag" or "sulfaplata" or "thermazene" or "uburn" or "ustionil").mp.
4. 1 and 2 and 3
5. Limit 4 to the English language

**Figure 1:**
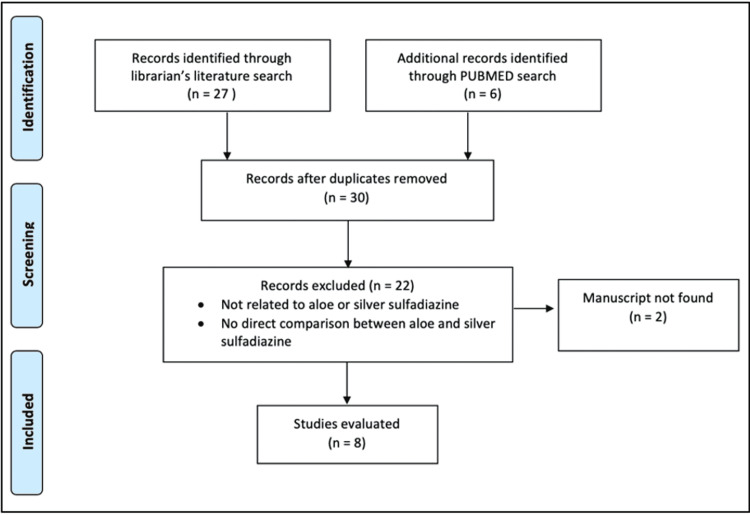
Inclusion and exclusion criteria for systematic review

A total of 30 articles were screened on the basis of the above search on the basis of title, abstract, and results, and seven full-text articles were reviewed [7,14,21-25]. To ensure that no relevant articles were missed in the search, the reference lists for each article were carefully screened. The items in the Preferred Reporting Items for Systematic Reviews and Meta-Analyses (PRISMA) 2015 checklist for systematic reviews were used as a guide for this review (Figure 1) [26]. Two independent reviewers used a standardized review form for data collection. Each of the articles meeting the inclusion criteria in Figure 1 underwent careful review and several data points were extracted including time to wound healing, pain scores after a week of treatment, and wound size after therapy.

A meta-analysis was performed for the number of days needed for healing. The pooled relative risk for days needed to heal is based on four studies using means and standard deviation. A random-effects model was used given the universality of the database search. Heterogeneity among studies was assessed using the χ2 test and I2 statistic. P-values are two-sided with statistical significance defined as p < 0.05. The risk of bias for each included study was assessed using the methodological index for non-randomized studies (MINORS) [27]. All statistical analyses and forest plots were performed using SAS statistical software version 9.4 (SAS Institute, Cary, NC).

Results

Study Selection

**Figure 2:**
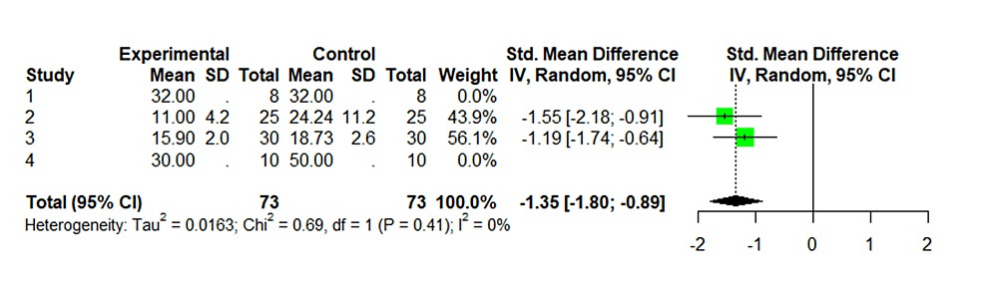
Random effect meta-analysis comparing time to healing of second- and third-degree burns using Aloe vera and silver sulfadiazine 1. Comparison of healing effect of Aloe vera extract and silver sulfadiazine in burn injuries in experimental rat model [25]. 2. Effectiveness of Aloe vera gel compared with 1% silver sulphadiazine cream as burn wound dressing in second degree burns [14]. 3. Aloe versus silver sulfadiazine creams for second-degree burns: a randomized controlled study [6]. 4. Comparative evaluation of Aloe vera in the management of burn wounds in guinea pigs [17].

A total of 33 articles were identified. After removing the duplicate articles, 30 remained between the medical librarian’s search and a separate PubMed search. Of these 30 articles, 22 were excluded because they were not related to AV or SSD, and/or there was no direct comparison in regards to efficacy between AV and SSD for the treatment of burns. Of the seven studies included, four of them were conducted in Iran [6,7,25,28], one in Pakistan [14], one in Turkey [23], and one in Puerto Rico (Table 2) [25]. When analyzing the number of days it took for the burn wound to heal, we observed that AV expedited healing in comparison to SSD (RR: -1.34, 95% CI: -1.8 to 0.9, p < 0.001, I2 = 0.0%) (Figure 2).

**Table 2:** Studies included in the systematic review SSD: silver sulfadiazine; AV: Aloe vera; TNF-α: tumor necrosis factor-alpha; IL-1β: interleukin 1 beta.

Year	Author	Title	Study design	Patient characteristics	Burn type	Treatments compared	Endpoint	Adverse outcomes	Conclusion
2017	Akgun et al. [23]	Evaluation of the wound healing potential of Aloe vera-based extract of Nerium oleander	Cohort study	24 Wistar albino male rats	2nd-degree partial thickness burn	Control group: no burn vs. burn alone group vs. burn with Nerium oleander leaf (NAE-8) vs. burn with 1% SSD	Serum malondialdehyde (MDA), glutathione (GSH), TNF-α, IL-1β levels, myeloperoxidase (MPO) activity, and percentages of DNA in the tail (%DNA_T_)	Vasocongestion = burn: moderate; Aloe: slight; SSD: moderate. Necrosis = burn: marked; Aloe: moderate; SSD: moderate. Inflammatory cell infiltration = burn: marked; Aloe: moderate; SSD: moderate	After NAE-8 treatment, the wound developed epithelization and reductions in the extent of necrosis and inflammatory cell infiltration. NAE-8-induced migration of fibroblasts
2014	Akhoondinasab et al. [25]	Comparison of healing effect of Aloe vera extract and silver sulfadiazine in burn injuries in experimental rat model	Randomized clinical trial	16 Wistar albino male rats	Deep 2nd-degree burns on the lower back and 3rd-degree burn on the upper back	Aloe vera gel vs. silver sulfadiazine	Epithelialization in 2nd and 3rd-degree burns: Aloe group showed greater epithelialization than the SSD group. Speed of healing: faster in the Aloe group	1 animal in the SSD group died	Wound healing was more visible in the Aloe vera group. The speed of healing in the Aloe vera group was faster than in the SSD group
2013	Shahzad et al. [14]	Effectiveness of Aloe vera gel compared with 1% silver sulphadiazine cream as burn wound dressing in second degree burns	Interventional comparative study	50 patients (26 males/24 females), mean age: 30	2nd-degree partial thickness burns	Aloe vera gel vs. 1% silver sulfadiazine	Average time for re-epithelialization = Aloe: 11 days; SSD: 24 days. Time taken for complete pain relief = Aloe: 21 days, SSD: 26 days	1/25 patients in the Aloe group had incomplete recovery. 6/25 patients in the SSD group had hypertrophic scar formation or the development of contractures. No differences in wound infections between both groups	Aloe vera gel promoted burn wound healing more effectively than SSD. Aloe vera tended to increase the rate of success in healing and the rate of epithelialization. Aloe is also more cost-effective than SSD
2012	Yunes et al. [28]	A herbal cream consisting of Aloe vera, Lavandula stoechas, and Pelargonium roseum as an alternative for silver sulfadiazine in burn management	Randomized, double-blinded clinical trial	111 patients. Herbal cream group (n = 56), mean age = 33.6 1%. Silver sulfadiazine group (n = 55), mean age = 37.4	Superficial 2nd-degree burns	Aloe vera gel + essential oils (Lavandula stoechas and Pelargonium roseum) vs. 1% silver sulfadiazine	Pain reduction: significantly greater pain reduction from baseline to seven days in the herbal cream group compared to the control group	One case of infection in the herbal cream group was cleared with the continuation of treatment	Herbal cream is superior to 1% SSD in the alleviation of pain and may serve as a natural alternative for the treatment of second-degree burns
2010	Hosseinimehr et al. [6]	Effect of Aloe cream versus silver sulfadiazine for healing burn wounds in rats	Comparative study	48 male Wistar rats	2nd-degree burns	Control (no topical agent) vs. base cream vs. Aloe cream (Aloe vera gel powder 0.5%) vs. 1% SSD	Mean wound size at 25 days = control: 5.5 ± 3 cm^2^, base: 4 ± 2.3 cm^2^, Aloe: 0.78 ± 1.3 cm^2^, SSD: 4.1 ± 3.6 cm^2^. At 25 days, the Aloe group had the smallest mean wound size	Bacteria were found in the control and base cream groups. SSD-treated wounds showed a lesser degree of re-epithelialization, fibrosis of the dermis with more ulceration, granulation of tissue formation, and inflammation	Aloe cream significantly increased re-epithelialization in burn wounds as compared to SSD
2009	Khorasani et al. [7]	Aloe versus silver sulfadiazine creams for second-degree burns: a randomized controlled study	Randomized controlled study	30 patients (25 M/5 F), mean age: 33	2nd-degree partial thickness burns of the hand or feet	Aloe cream (Aloe vera gel powder 0.5%) vs. SSD	Time to burn healing = Aloe: 16 days, SSD: 19 days	None	The rate of re-epithelialization and healing of the partial thickness burns was significantly faster in the site treated with Aloe than in the site treated with SSD
1988	Rodriguez-Bigas et al. [17]	Comparative evaluation of Aloe vera in the management of burn wounds in guinea pigs	Comparative study	40 male Hartley guinea pigs	Full-thickness burns	Silver sulfadiazine vs. Aloe vera gel extract vs. salicylic acid cream vs. plain gauze	Avg. time to complete healing = control: 50 days, SSD: 47 days, salicylic acid: 45 days, Aloe: 30 days	The use of salicylic acid cream alone failed to provide a significant bacteriostatic effect. The control group had greater than 10^5^ bacteria per gram of tissue	Aloe gel extract permits faster healing of burn wounds compared to other topical treatments. Wound bacterial counts were effectively decreased by SSD and AV

Risk of Bias

Table 3 presents the quality assessment of the included studies using the MINORS tool. The mean MINORS score across all included studies was 20.3 (range: 19-22) out of 24. All studies included prospectively collected data with study and control groups. The aims were clearly stated and the outcomes were free of bias. Sensitivity analysis was omitted due to the low number of included studies.

**Table 3:** Methodological index for non-randomized studies (MINORS)

Study	MINORS criteria												
	Stated aim	Consecutive patients	Prospective data collection	Appropriate endpoints	Unbiased endpoint	Follow-up period	Lost to follow-up	Calculation of sample size	Control group	Contemporary groups	Baseline equivalence of groups	Statistical analysis	Total
Akgun et al. (2017) [23]	2	2	2	1	2	2	2	0	2	2	2	0	19
Shahzad et al. (2013 ) [14]	2	2	2	2	2	2	2	0	2	2	2	2	22
Hosseinimehr et al. (2010) [6]	2	2	2	1	2	2	2	0	2	2	2	0	19
Khorasani et al. (2009) [7]	2	2	2	2	2	2	2	0	2	2	2	2	22
Rodriguez-Bigas et al. (1988) [17]	2	2	2	2	2	2	2	0	2	2	2	1	21

Study Comparisons: Animal Studies

A cohort study conducted in 2017 by Akgun et al. also used Wistar albino rats to compare wound healing outcomes for induced partial-thickness second-degree burns [23]. A total of 24 rats were evenly split into four groups: a control group (no burn), a burn alone group, a burn group treated with an AV-based extract of the Nerium oleander leaf (NAE-8), and a burn group treated with 1% SSD. Each group received the corresponding topical treatment twice a day for the duration of 14 consecutive days. The levels of serum malondialdehyde (MDA), glutathione (GSH), TNF-α, and interleukin 1 beta (IL-1β) were analyzed in each group, as well as myeloperoxidase (MPO) activity and percentages of DNA in the tail (%DNAT). MDA levels in the control group were 15.23 ± 1.74 nmol/g tissue. In comparison to the control group, MDA levels in the burn group, SSD group, and NAE-8 group were 185.38 ± 17.55 nmol/g, 135.49 ± 22.83 nmol/g, and 47.07 ± 12.71 nmol/g, respectively. The decrease in MDA levels was statistically significant between the burn group and the burn + NAE-8-treated group (p < 0.001). Whereas, the decrease in MDA levels between the burn group and the burn + SSD group was not statistically significant. The increase in glutathione levels in the burn + NAE-8 group compared to the burn group was statistically significant (p < 0.001). The increase in glutathione levels in the SSD group compared to the control group was also statistically significant (p < 0.05). Additionally, the decrease of MPO activity in the burn + NAE-8 group compared to the burn group was statistically significant (p < 0.01). Whereas the decrease in MPO activity between the burn group and the burn + SSD group was not statistically significant. In regards to the reduction of the proinflammatory cytokine, TNF-α, both the NAE-8 and SSD group similarly decreased their levels compared to the burn group (p < 0.01 for both groups). Both the NAE-8 and SSD groups similarly decreased IL-1β levels in comparison to the burn group (p < 0.001 for both groups). The decrease of %DNAT in the burn + NAE-8 group compared to the burn group was statistically significant (p < 0.01). The %DNAT is the only measure where the SSD group showed slightly superior results over the NAE-8 group. The authors concluded that NAE-8 has potent antioxidant and anti-inflammatory effects, as well as DNA repair capacity that significantly reversed the thermal injury-induced changes in MDA, GSH, MPO, TNF-α, IL-1β, and %DNAT levels.

In 2014, Akhoondinasab et al. conducted a randomized clinical trial on Wistar albino rats with induced deep second-degree burns on the lower back and third-degree burns on the upper back [25]. The 16 rats were split into two different groups; eight rats were treated with AV extract and the other eight with SSD. Burns were dressed daily in both groups and digital photography was used for 32 days to assess the efficacy of each treatment. The wound healing between groups for second-degree burns was significantly better in the Aloe group (p < 0.005) in 29 of 32 days. The wound healing between groups for third-degree burns was also significantly better and significantly shorter in the Aloe group (p < 0.005) in 29 of 32 days. Furthermore, epithelialization was more evident in the Aloe-treated group. There were no complications in the Aloe group; however, one rat in the SSD-treated group died. The authors of this study concluded that wound healing for second-degree burns, including the speed of healing, was superior in the Aloe group.

A comparative study conducted in 2010 by Hosseinimehr et al. studied 48 Wistar rats with induced second-degree burns [21]. The rats were split into four groups, with 12 rats in each group. There was a control group (no topical agent applied), base cream-treated group (no effective agent), Aloe cream-treated group (0.5% AV gel powder), and 1% SSD-treated group. Wounds were treated twice daily beginning 24 hours post-burn injury. Mean wound size was measured at seven, 10, 14, 20, and 25 days after the burn. The mean wound sizes at 25 days were 5.5 ± 3 cm2, 4 ± 2.3 cm2, 4.1 ± 3.6 cm2, and 0.78 ± 1.3 cm2 in the control, base, SSD, and Aloe groups, respectively. The comparison of the Aloe cream group to all other groups was statistically significant (p < 0.05). In terms of antibacterial activity, bacteria were found in the skin specimens for the control and base cream groups, but not in the Aloe and SSD groups. However, compared to the Aloe group, the SSD-treated burns showed a lesser degree of re-epithelialization, more inflammation, and increased fibrosis and ulceration of the dermis. Based on the results of this study, the authors concluded that not only were the wounds significantly smaller in the Aloe group compared to the other groups but also treatment with Aloe was able to notably increase re-epithelialization of the burn wounds.

The earliest article reviewed was a comparative study conducted in 1988 by Rodriguez-Bigas et al. on 40 Hartley guinea pigs with induced third-degree burns [17]. The 40 guinea pigs were split into four groups: 10 in the SSD-treated group, 10 in the AV gel extract-treated group, 10 in the salicylic acid (SA) cream-treated group, and 10 in a control group that only received a plain gauze occlusive dressing. On day six post-burn, all animals in the control group had greater than 105 bacteria per gram of tissue. The SA cream group did not provide a significant bacteriostatic effect; 80% of the animals had greater than 105 bacteria per gram of tissue (p = 0.450). However, both the SSD and Aloe-treated groups demonstrated significant antimicrobial effects, with 60% of animals having less than 105 bacteria per gram of tissue (p = 0.015). The mean time to complete wound healing was 50, 45, 47, and 30 days for the control group, SA cream group, SSD group, and Aloe group, respectively. Compared to the control group, only the Aloe group significantly expedited wound healing (p < 0.02).

Study Comparisons: Human Studies

In 2013, an interventional comparative study by Shahzad et al. was conducted on 50 patients to assess the effectiveness of AV gel and 1% SSD on wound healing of second-degree burns [14]. A total of 25 patients were placed in the AV group and 25 patients were placed in the SSD group. As a precaution, all patients were started on a third-generation cephalosporin. The wound dressing was changed twice daily until the wound was fully healed. The mean time for wound re-epithelialization in the Aloe group was 11 ± 4.18 days and 24.24 ± 11.16 days in the SSD group (p < 0.0001). Time taken for complete pain relief was 21 days in the Aloe group and 26 days in the SSD group (p = 0.01). There were no differences in wound infections between the two groups: three in the Aloe group and four in the SSD group. However, in terms of healing outcomes, six out of 25 patients in the SSD group experienced hypertrophic scar formation or the development of contractures, whereas only one out of 25 patients in the Aloe group had an incomplete recovery. The authors of this study concluded that compared to SSD, the burn wounds treated with Aloe showed earlier wound epithelialization and earlier pain relief. Moreover, another advantage of AV gel was that it is more cost-effective than SSD.

A similar study was conducted by Khorasani et al. in 2009 [7]. A total of 30 patients were enrolled in a randomized controlled study to assess the effectiveness of AV cream (0.5% AV gel powder) and 1% SSD on wound healing of second-degree burns. Each patient had similar types of second-degree burns at two sites on different parts of the body. Thus, the same patient received treatment with either Aloe cream or SSD on each of the wounds. The mean healing time was significantly shorter for the Aloe group at 15.9 ± 2 days versus 18.73 ± 2.65 days for the SSD group (p < 0.0001). Both sites were negative for microbial contamination on days three, seven, and 13. The authors concluded that AV cream promoted better wound healing with smaller lesions and shorter healing times than SSD.

A randomized double-blinded clinical trial was conducted by Yunes et al. in 2012, which compared the efficacy of an AV-based herbal cream with 1% SSD on the pain reduction of second-degree burns [28]. The herbal cream consisted of AV gel and the essential oils of *Lavandula stoechas* and *Pelargonium roseum*. A total of 56 of 111 patients were randomized to the herbal cream group and the other 55 were assigned to the SSD group. The results of the study demonstrated that the pain severity at 14 days was significantly reduced in both groups compared to baseline (p < 0.001). However, there was a greater reduction of pain from baseline to the seven and 14-day mark in the herbal cream group (p = 0.014 and p = 0.05). The difference in skin dryness between the two groups was not statistically significant. One case of infection was reported in the herbal cream group; however, it cleared up with the continuation of treatment. The authors of this clinical trial concluded that the herbal cream was superior to SSD in the alleviation of pain for superficial second-degree burns.

Discussion

To the best of our knowledge, this is the first systematic review conducted that compares the efficacy of AV gel and SSD for burn wound healing. Existing literature demonstrates that AV gel seems to be efficacious in the treatment of second-degree burns with no or less toxicity than SSD [6,7,14,23,25,28]. Moreover, AV is a less expensive and more accessible option than SSD, making it a more cost-effective and convenient treatment option. SSD is currently the standard of care for topical treatment of second- and third-degree burns [3]; however, some existing literature demonstrates that SSD may cause delays in wound healing [5,6]. A study conducted by Muller et al. [5] demonstrated delayed wound contraction caused by SSD that was reversed by the addition of AV gel. In this study, it would appear that healing time was shortest in the SSD/AV group and longest in the 1% SSD group. Moreover, other adverse side effects reported for SSD included the generation of black scars, limited wound penetration, hypersensitivity, renal toxicity, and leukopenia [6,28]. Therefore, SSD is not recommended for long-term use. Finally, the use of SSD for partial-thickness burn wounds results in an increased cost of patient care [6].

The studies analyzed demonstrate a statistically significant benefit to using AV over SSD for wound healing, including faster time to wound re-epithelialization [6,7,14,17,25], a reduction in pro-inflammatory cytokines [23], and greater pain relief [28].

Our systematic review reinforces the time-to-healing advantages that AV gel offers for the management of second- and third-degree burns. Additionally, AV gel is a more accessible and less expensive treatment option than SSD, which is especially important considering that the majority of all burns occur in low- and middle-income countries. Although the relative risk favors the use of AV for burn healing, our review would benefit from the incorporation of more trials comparing both treatment options. Further trials may aid the support of using AV for the management of high-risk burns.

## Conclusions

SSD is currently regarded as the gold standard for topical burn treatment, frequently utilized for second- and third-degree burns. However, growing concerns over side effects associated with its use as well as cost prohibitions are the impetus for finding an alternative or supplemental solution. Our systematic review and meta-analysis demonstrate the time to healing with the use of AV gel shows earlier wound epithelialization, pain relief, no or fewer side effects, and equally efficacious antibacterial properties when compared to SSD. Increased consideration and emphasis should be placed on the use of AV gel to aid the healing of second- and third-degree burns. Further trials comparing burn wound healing parameters between AV and SSD are needed.
